# Social justice design and implementation in library and information science

**DOI:** 10.29173/jchla29663

**Published:** 2023-04-01

**Authors:** Stacy Brody

**Affiliations:** Reference and Instruction Librarian Himmelfarb Health Sciences Library The George Washington University Washington, D.C., U.S.A.

Mehra B, editor. **Social justice design and implementation in library and information science**. New York: Routledge; 2021. Softcover: 332p. ISBN 978-0-367-65382-8. Price: USD$48.95. Available from: https://www.routledge.com/Social-Justice-Design-and-Implementation-in-Library-and-Information-Science/Mehra/p/book/9780367653828

*Social justice design and implementation in library and information science* presents a diversity of cases pertinent to librarianship, information science research, and library and information science (LIS) education. Editor Dr. Bharat Mehra is the EBSCO Endowed Chair in Social Justice and Professor in the School of Library & Information Studies at the University of Alabama. Dr. Mehra earned his PhD in Library and Information Science from the University of Illinois at Urbana-Champaign after also earning degrees in South Asian and Middle Eastern Studies and Landscape Architecture. This background sheds light on the selection of topics in this collection, including a chapter discussing cultural heritage studies as applied to built spaces (chapter 9).

I volunteered to review this book because of my interest in the topic. I hoped the text would help me generate ideas for my workplace’s Diversity, Equity, and Inclusion Committee and Hiring Practices Evaluation Taskforce. As an instruction librarian and liaison to pre-clinical medical school courses, I was interested to see how social justice discussions in health professional education compared to similarly focused conversations in my own professional community. I acknowledge my own bias for cases in settings most relevant to my own workplace.

The case studies in this text vary greatly, not only in terms of topics and settings, but also in their presentation and seemingly intended audiences. Cases are organized into five sections: (I) emerging new responsibilities, (II) reflective case practices, (III) reaching out: new research approaches and strategies, (IV) transforming LIS education, and (V) instruments of action and change. These categories try to cover the broad range of questions relevant to social justice.

Notably, authors take a variety of approaches in presenting their work. Some case studies follow a traditional structure: background, literature review, and statement of purpose. Reflecting a familiar outline, these chapters appealed to me most. Chapter 4, for instance, presents a structured case study of a public library involved in food justice. Though the setting (rural public libraries) and the intervention (food justice) differ greatly from my own work, I was able to relate to themes described by the authors. Not all chapters provide such entrée points.

Other cases are grounded in theory. While interested in the theory of our profession, I found these chapters less practical and relevant to my role as a reference and instruction librarian in an academic medical center. They may be more pertinent to the work of information science researchers and LIS educators.

Finally, some contributors take a narrative approach. This documentation is crucial as these stories are often lost. In the foreword, Paul T. Jaeger, Co-Director of the Information Policy and Access Center (iPAC) and former MLIS program Co-Director at the College of Information Studies, University of Maryland, describes the importance of the current collection in documenting our profession’s legacy.

Our professional roles and research interests are broad and their intersections with social justice are many, as demonstrated in the diversity of content within this text. I appreciated learning about the work of librarians and information professionals in this space, but I wanted more tie-ins to larger contexts, descriptions of impact, lessons learned, or most useful for me, practical tips and potential applications to other settings.

Future printings should correct typos and copyediting mistakes. I was often distracted from the content by these errors. Additionally, I found it difficult to switch between chapters, as some used dense, jargon-laden text, whereas others used plain language and storytelling style.

Overall, I found the stories illuminating and inspiring. I was able to relate in some ways, even to librarians working in other contexts. Personally, I am interested in seeing how professional schools adapt and update curricula to meet the ever-changing needs of the workplace, as described by cases in section IV, transforming LIS education. With its expansive view of social justice in library and information science, this text would be a good addition to collections serving library and information science schools or degree programs. Yet, even while I acknowledge the importance of its topic, I would not recommend this book for purchase by a health sciences library.

